# Maternal drinking and smoking. Can it explain the exceptional academic performance of LBOTE children? A preliminary analysis

**DOI:** 10.1186/s13104-021-05544-5

**Published:** 2021-04-16

**Authors:** Louisa Gibson, Melanie Porter

**Affiliations:** Faculty of Medicine, Health and Human Sciences, Department of Psychology, Macquarie University, Balaclava Road, North Ryde, Sydney, NSW 2109 Australia

**Keywords:** Academic achievement, Non-English speaking background, Drinking, Smoking, Breastfeeding, Pregnancy

## Abstract

**Objective:**

Although children from language backgrounds other than English (LBOTE) may be disadvantaged in English-reliant exams, they outperform children from an English language background (ELB) on many Australian National Assessment Program–Literacy and Numeracy (NAPLAN) assessments. Maternal alcohol and tobacco use during pregnancy and/or breastfeeding have been associated with poorer cognitive and academic performance. Using data from the Growing Up in Australia Study, this paper aimed to identify demographic, lifestyle, and prenatal and perinatal risk differences related to maternal tobacco and alcohol use between LBOTE and ELB groups, as a first step in trying to understand the academic performance differences.

**Results:**

Only data from breastfed babies was included in the current analyses. Although LBOTE children were disadvantaged in several demographic areas, their NAPLAN performance was the same or superior to ELB children across all Grade 3 and 5 NAPLAN assessments. The LBOTE group were, however, breastfed for longer, and their mothers smoked fewer cigarettes and drank less alcohol on fewer occasions throughout their pregnancy. The LBOTE mothers also had lower or less risky patterns of alcohol consumption while breastfeeding. The longer breastfeeding duration of LBOTE children combined with lower maternal use of alcohol and cigarettes during pregnancy and/or breastfeeding may partially contribute to their exceptional NAPLAN performance.

## Introduction

Children from language backgrounds other than English (LBOTE) perform better than children from an English language background (ELB) in many of the Australian National Assessment Program–Literacy and Numeracy (NAPLAN) assessments [1, 2]. While it has been suggested that this may be due to inadequacies in the NAPLAN LBOTE measure [3, 4], it is possible that health, family, lifestyle, environmental or socio-economic status (SES) factors may be at least partly responsible.

The NAPLAN assessments are a series of English-reliant annual tests in reading, writing, language conventions (spelling, grammar and punctuation) and numeracy. These tests are given to all Australian children in grades 3, 5, 7 and 9 [1].

Given that greater English proficiency has been associated with superior academic performance [5], LBOTE children may be disadvantaged. Despite this expectation, it has been repeatedly observed that LBOTE children perform better on NAPLAN tests of writing, spelling, grammar and numeracy [2]. Although it is possible that disadvantaged LBOTE children may tend to stay home on the day of NAPLAN assessments, Creagh [3, 4] suggested that the superior performance of LBOTE students is due to the way LBOTE status is assigned being an inadequate measure of English proficiency.

If usual ways of measuring LBOTE status are biased in favour of LBOTE children, or fail to measure English proficiency, LBOTE children would be expected to outperform ELB children across different education systems that utilise similar LBOTE measures. While LBOTE children in the United Kingdom did outperform ELB children for the first time in 2017 [6], LBOTE students in the United States [7] and Canada [8] perform more poorly on academic assessments. This suggests that other factors may contribute to the superior performance of LBOTE students.

Children’s academic achievement may be impacted by many cognitive, physiological, lifestyle, environmental and SES variables. Socio-economic factors such as lower parental education and income have been shown to negatively impact academic achievement in children [9], while homework completion has been associated with improved outcomes [10]. Higher parental expectations and receiving assistance with homework have also been associated with greater academic achievement [11]. Additionally, older age [12], and higher birth weight [13], are all related to greater cognitive ability, which in itself is associated with better academic outcomes [14]. Sex differences in individual cognitive abilities have also been postulated [15]. Additionally, while there is conflicting evidence [16], longer breastfeeding duration has further been associated with increased cognitive ability [17].

Prenatal maternal alcohol and tobacco intake have also been found to negatively impact children’s academic performance [18, 19]. While no changes in developmental health outcomes have been observed [20], dose-dependent reductions in cognitive and academic outcomes have been found in children of mothers who consumed alcohol while breastfeeding [21, 22]. This suggests that maternal alcohol consumption or tobacco smoking while breastfeeding may be additional factors that contribute to differences in academic achievement between LBOTE and ELB children.

Using data from Growing Up in Australia: The Longitudinal Study of Australian Children (LSAC) [23], the current study was a preliminary analysis describing the differences in lifestyle, demographic, SES and maternal drinking and smoking habits between LBOTE and ELB groups in a sample of Australian breastfed babies. The primary goal was to identify demographic, lifestyle, and prenatal and perinatal risk differences related to maternal tobacco and alcohol use between the two groups as a first step in trying to understand the academic performance differences. It was anticipated that differences would be observed between the two groups that may help to explain the superior performance of LBOTE students, and provide a basis for future research.

## Main text

### Method

#### Study design, data source and study cohort

The study cohort has been described previously [20–22]. Briefly, data was sourced from LSAC. The study cohort comprised 5107 infants and caregivers from LSAC who were recruited in 2004 (average age of 9 months) and followed over time every two years in waves [24]. Six data waves were available for analyses. Only babies who were breastfeeding at study entry (Wave 1) with data available from their biological mothers were included in the analyses.

#### Study variables

The sample was divided into LBOTE and ELB children. All other variables were compared between these two groups. The language background of children was categorised as LBOTE if the primary language spoken at home at study entry was not English, and ELB if the primary language was English. Other study variables described previously [21, 22] included:

##### Academic outcome variables

- Grades 3 and 5 NAPLAN reading scores
- Grades 3 and 5 NAPLAN writing scores
- Grades 3 and 5 NAPLAN spelling scores
- Grades 3 and 5 NAPLAN grammar and punctuation scores
- Grades 3 and 5 NAPLAN numeracy scores

##### Demographic variables

- Sex of child
- Birth weight (grams)
- Combined family annual income $AUD (higher score indicates lower income)
- Maternal education
- Breastfeeding duration (days)
- Age at Grade 3 NAPLAN test
- Age at Grade 5 NAPLAN test

##### Tobacco and alcohol use variables

- Maternal average daily cigarettes smoked while pregnant
- Maternal average daily cigarettes smoked while breastfeeding
- Maternal alcohol consumed while breastfeeding (mother’s total modified version of The Alcohol Use Disorders Identification Test Alcohol Consumption Questions; AUDIT-C [25, 26] score at wave 1)
- Days per week mother drank alcohol during first trimester of pregnancy
- Days per week mother drank alcohol during second trimester of pregnancy
- Days per week mother drank alcohol during third trimester of pregnancy
- Average number of alcoholic drinks mother consumed on each drinking occasion during pregnancy

Additional homework and tutoring variables are described in Table 1.

**Table 1:** Description and response options for variables included in analyses

Variable	Description	Response options and values
Homework frequency (Wave 4)	How often does study child do homework?	1: Daily
		2: A few times a week
		3: Once a week
		4: A few times a month
		5: Once a month
		6: Less than once a month
		7: Never
Homework help (Wave 4)	During this school year, how often have you or another family member (or adult in the household) helped study child with his/her homework?	1: Daily
		2: A few times a week
		3: Once a week
		4: A few times a month
		5: Less often
Tutoring (Wave 4)	In the last 12 months has the study child received any additional help or tutoring from anyone outside the household?	1: Yes
		2: No
		(− 2: Don’t know-coded as missing data)
Tutoring frequency (Wave 4)	How often?	1: More than once a week
		2: Once a week
		3: Less than once a week
		(− 2: Don’t know-coded as missing data)
Homework hours (Wave 5)	In an average week, how many hours does study child spend on homework outside of school?	Number of hours
Homework help (Wave 5)	During this school year, how often did someone in this household help study child with homework?	1: 5 or more days a week
		2: 3 or 4 days a week
		3: 1 or 2 days a week
		4: Less than once a week
		5: Never
Tutoring (Wave 5)	In the last 12 months has study child received any additional help or tutoring from anyone outside the household?	1: Yes
		2: No
		(− 2: Don’t know-coded as missing data)
Tutoring frequency (Wave 5)	How often?	1: More than once a week
		2: Once a week
		3: Less than once a week
		(− 2: Don’t know-coded as missing data)
Homework hours (Wave 6)	In an average week, how many hours does study child spend on homework outside of school?	Number of hours
Homework help (Wave 6)	During this school year, how often did someone in this household help study child with his/her homework?	1: 5 or more days a week
		2: 3 or 4 days a week
		3: 1 or 2 days a week
		4: Less than once a week
		5: Never
Tutoring (Wave 6)	In the last 12 months has study child received any additional help or tutoring from anyone outside the household?	1: Yes
		2: No
		(− 2: Don’t know-coded as missing data)
Tutoring frequency (Wave 6)	How often?	1: More than once a week
		2: Once a week
		3: Less than once a week
		(− 2: Don’t know-coded as missing data)

### Statistical analyses

Data was analysed using IBM SPSS version 24 (α = 0.05, 2-tailed). Due to data skew, variables were analysed using Kruskal–Wallis H tests, with the exception of dichotomous variables that were analysed using a one-way analysis of variance (ANOVA). To ensure that the academic performance between LBOTE and ELB children was not an artefact of lower SES LBOTE children being kept home on the day of assessment, two-way analyses of variance were used to determine maternal education and combined family income interaction effects between NAPLAN completers and non-completers across LBOTE and ELB groups. Corrections for multiple comparisons were not made, since the purpose of the study was to provide a brief and preliminary descriptive analysis of the sample between groups.

## Results

It was found that LBOTE children performed better than ELB children on grade 3 NAPLAN writing and spelling tests, as well as grade 5 NAPLAN spelling tests. It was also observed that LBOTE children were younger at the time of grade 3 NAPLAN tests, were from lower income families and were of lower birthweight than ELB children. Mothers of LBOTE children smoked fewer cigarettes during pregnancy and while breastfeeding, and had lower alcohol drinking scores while breastfeeding. They also drank alcohol on fewer days per week during each trimester of pregnancy and consumed less alcohol on each occasion. No other differences between LBOTE and ELB children were observed (Table 2a–d).

**Table 2:** Results of ANOVA and Kruskal–Wallis H tests between LBOTE and ELB groups

Variable	LBOTE		ELB		Statistic	p-value
	N	M (SD)	N	M (SD)		
(a) Academic outcome variables						
Grade 3 NAPLAN reading scores	137	449.28 (89.86)	1379	446.71 (93.45)	0.23^b^	0.63
Grade 3 NAPLAN writing scores	137	442.38 (59.53)	1372	426.30 (61.22)	10.50^b^	< 0.01
Years 3 NAPLAN spelling scores	137	450.78 (91.44)	1372	420.09 (77.15)	19.56^b^	< 0.001
Grade 3 NAPLAN grammar and punctuation scores	137	461.09 (97.80)	1372	446.30 (90.16)	1.61^b^	0.21
Grade 3 NAPLAN numeracy scores	135	424.48 (84.01)	1376	415.24 (72.24)	0.93^b^	0.34
Grade 5 NAPLAN reading scores	113	523.74 (81.73)	1257	529.52 (81.65)	0.31^b^	0.58
Grade 5 NAPLAN writing scores	112	492.97 (66.66)	1249	482.38 (65.38)	3.61^b^	0.06
Grade 5 NAPLAN spelling scores	112	535.73 (76.60)	1257	505.73 (71.96)	14.28^b^	< 0.001
Grade 5 NAPLAN grammar and punctuation scores	112	540.00 (91.40)	1257	528.35 (83.39)	1.51^b^	0.22

Variable	LBOTE		ELB		Statistic	p-value
	N	M (SD)	N	M (SD)		
(b) Demographic variables						
Sex (male/female)	215	1.53 (0.50)	1790	1.50 (0.50)	0.81^a^	0.37
Birth weight (grams)	213	3399.42 (523.14)	1780	3478.84 (539.92)	5.65^b^	0.02
Combined family annual income $AUD *	196	6.67 (2.93)*	1691	5.53 (2.56)*	26.22^b^	< 0.001
Maternal education	213	6.22 (1.78)	1788	6.30 (1.49)	0.01^b^	0.93
Breastfeeding duration (days)	200	255.98 (202.93)	1546	228.87 (203.93)	4.60^b^	0.03
Age at Grade 3 NAPLAN test (months)	141	101.77 (4.16)	1429	102.68 (4.27)	4.17^b^	0.04
Age at Grade 5 NAPLAN test (months)	119	125.61 (3.90)	1293	126.09 (3.57)	1.34^b^	0.25

Variable	LBOTE		ELB		Statistic	p-value
	N	M (SD)	N	M (SD)		
(c) Tobacco and alcohol use variables						
Maternal average daily cigarettes smoked while pregnant	143	0.14 (1.07)	1523	0.66 (2.65)	9.29^b^	< 0.01
Maternal average daily cigarettes smoked while breastfeeding	165	0.45 (2.02)	1590	1.06 (3.79)	2.87^b^	0.09
Days per week mother drank alcohol during 1st trimester of pregnancy	150	0.06 (0.35)	1584	0.18 (0.74)	5.13^b^	0.02
Days per week mother drank alcohol during 2nd trimester of pregnancy	150	0.04 (0.26)	1582	0.22 (0.76)	10.25^b^	< 0.01
Days per week mother drank alcohol during 3rd trimester of pregnancy	150	0.05 (0.28)	1581	0.25 (0.87)	8.02^b^	0.01
Average number of alcoholic drinks mother consumed on each drinking occasion during pregnancy	150	0.19 (0.41)	1579	0.48 (0.54)	41.47^b^	< 0.001
Maternal alcohol consumed while breastfeeding	151	3.09 (2.48)	1338	5.83 (2.29)	126.69^b^	<0.001

Variable	LBOTE		ELB		Statistic	p-value
	N	M (SD)	N	M (SD)		
(d) Homework and tutoring variables						
Homework frequency (Wave 4)	178	1.38 (0.74)	1392	1.39 (0.64)	0.36^b^	0.55
Homework help (Wave 4)	178	1.45 (0.74)	1392	1.44 (0.71)	0.04^b^	0.84
Tutoring (Wave 4)	178	1.88 (0.32)	1389	1.89 (0.31)	0.14^a^	0.71
Tutoring frequency (Wave 4)	20	1.70 (0.57)	151	1.81 (0.68)	0.33^b^	0.57
Homework hours (Wave 5)	164	2.70 (2.11)	1333	2.48 (1.80)	0.82^b^	0.37
Homework help (Wave 5)	164	2.25 (1.04)	1333	2.12 (0.99)	2.40^b^	0.12
Tutoring (Wave 5)	168	1.89 (0.32)	1387	1.84 (0.37)	2.46^a^	0.12
Tutoring frequency (Wave 5)	19	1.63 (0.50)	219	1.85 (0.63)	2.05^b^	0.15
Homework hours (Wave 6)	146	2.77 (1.99)	1164	2.74 (1.90)	< 0.001^b^	0.99
Homework help (Wave 6)	146	2.47 (1.03)	1164	2.64 (1.01)	4.23^b^	0.04
Tutoring (Wave 6)	153	1.79 (0.41)	1239	1.84 (0.37)	1.92^a^	0.17
Tutoring frequency (Wave 6)	32	1.88 (0.55)	203	1.87 (0.57)	0.01^b^	0.93

*LBOTE* Language Background other than English, *ELB *English Language Background

^*^Higher scores indicate lower income; ^*a*^F value; ^*b*^χ^2^ value

Results indicated that Grade 3 and 5 NAPLAN non-completers were from families with lower levels of maternal education and lower family incomes (F = 15.77–24.59, p ˂ 0.001). For Grade 3 NAPLAN completers and non-completers, the magnitude of these differences did not vary between LBOTE and ELB groups (F = 1.78–3.87, p = 0.05–0.18). In contrast to this, the magnitude of the difference in maternal education (F = 6.07–7.12, p = 0.01), but not family income (F = 0.25–0.76, p = 0.38–0.55), was greater in the LBOTE group for grade 5 NAPLAN completers and non-completers.

## Discussion

The academic performance of breastfeeding LBOTE children was the same or superior to ELB children across all NAPLAN tests. Separate analyses also found that LBOTE children were of lower birthweight, from families of lower income, of the same or younger age at the time of NAPLAN tests, and received the same or less amount of homework, homework assistance, and tutoring. There were no differences between LBOTE and ELB children regarding sex, level of maternal education or the number of cigarettes their mothers smoked while breastfeeding. Contrastingly, LBOTE children were breastfed for longer than ELB children, and their mothers smoked fewer cigarettes during pregnancy. Mothers of LOBTE children also drank less alcohol on fewer occasions throughout their pregnancy, and had lower or less risky patterns of alcohol consumption while breastfeeding.

Non-completing NAPLAN children were found to be from families with lower levels of maternal education and family income. The magnitude of this difference did not vary between LBOTE and ELB groups in Grade 3, or regarding Grade 5 income. There was, however, a greater difference in the magnitude of the difference among LBOTE children in relation to maternal education in Grade 5.

These findings are somewhat contradictory. The superior performance of LBOTE children across some of the NAPLAN assessments has been previously observed [2], and lack of sex differences or maternal smoking during breastfeeding is unsurprising [15, 21, 22]. Contrastingly, however, the lower birthweight and family income [9, 13], younger age at the time of grade 3 NAPLAN assessments [12], and lack of additional homework, homework assistance or tutoring [10, 11], would be expected to disadvantage LBOTE children. Even in NAPLAN assessments where LBOTE children matched the performance of ELB children, findings are still unexpected given their LBOTE status [5].

Creagh [3, 4] suggested that the measurement of the LBOTE category itself is flawed, which may account for the superior or matched performance of LBOTE children on NAPLAN assessments. Additionally, and despite observed disadvantages, it is also possible that LBOTE children have some advantages that may also contribute to their performance. For example, the LBOTE group were breastfed for longer. Longer breastfeeding duration has been associated with better cognitive outcomes [17]. Likewise, the lower prenatal maternal tobacco and alcohol use [18, 19], and lower or less risky maternal use of alcohol while breastfeeding in LBOTE children [21, 22], has been previously associated with improved cognitive and academic outcomes.

While it would be premature to directly attribute the NAPLAN performance of LBOTE children to these breastfeeding and tobacco and alcohol measures, it is nonetheless noteworthy that these were the only variables in which LBOTE children were advantaged over ELB children. As such, it is possible that longer breastfeeding duration, lower prenatal maternal tobacco and alcohol use, and lower or less risky maternal use of alcohol while breastfeeding may partially contribute to the achievement of LBOTE children in NAPLAN assessments. This suggests that maternal factors may impact the academic performance of children at a later age.

Alternatively, LBOTE children who complete NAPLAN may be from more advantaged families than LBOTE children who do not complete NAPLAN. The NAPLAN completers were found to be from higher income families with greater maternal education regardless of language status. Since, however, the magnitude of this difference was only greater in LBOTE children when examining Grade 5 income, and Grade 3 LBOTE children also had significant academic achievement, this theory does not sufficiently explain the findings.

The academic performance of LBOTE children was the same or superior to ELB children across all Grade 3 and 5 NAPLAN assessments. This was despite LBOTE children being of lower birthweight, lower family income, of the same or younger age at the time of NAPLAN assessments, and receiving the same or less amount of homework, homework assistance, and tutoring. Additionally, LBOTE children breastfed for longer than ELB children, and their mothers smoked fewer cigarettes during pregnancy. Mothers of LOBTE children also drank less alcohol on fewer occasions throughout their pregnancy, and had lower or less risky patterns of alcohol consumption while breastfeeding. Since these factors have all been previously associated with improved cognitive or academic performance, they may partially help to explain the academic performance of LBOTE children. Future research should seek to clarify this, and more comprehensively assess the contribution of breastfeeding duration and maternal use of alcohol and tobacco on academic performance.

## Limitations

There were several limitations. These were simple analyses without correction for multiple comparisons. Additionally, using a parametric test to analyse differences between NAPLAN completers and non-completers may introduce error, given mild to moderate skew. Many of the variables relied on self-report measures, and biological confirmation of substance use was not undertaken. It was also not possible to combine alcohol and smoking variables due to measurement differences. Caution must therefore be used when interpreting findings. Future research should seek to conduct more complex analyses and identify the magnitude to which these breastfeeding and alcohol and tobacco variables may be contributing to the academic performance of LBOTE children.

## Data Availability

The dataset analysed during the current study is available by application to LSAC via the instructions available here: https://growingupinaustralia.gov.au/data-and-documentation

## Abbreviations

LBOTE: Language backgrounds other than English
ELB: English language background
NAPLAN: National Assessment Program–Literacy and Numeracy
SES: Socio-economic status
LSAC: Growing Up in Australia: The Longitudinal Study of Australian Children
AUDIT-C: The Alcohol Use Disorders Identification Test Alcohol Consumption Questions
ANOVA: Analysis of variance
